# α-Synuclein upregulates bim-mediated apoptosis by negatively regulating endogenous GCN5

**DOI:** 10.18632/aging.204353

**Published:** 2022-10-27

**Authors:** Shofiul Azam, In-Su Kim, Dong-Kug Choi

**Affiliations:** 1 Department of Applied Life Science, Graduate School, BK21 Program, Konkuk University, Chungju 27478, Korea; 2 Department of Biotechnology, College of Biomedical and Health Science, Research Institute of Inflammatory Disease (RID), Konkuk University, Chungju 27478, Korea

**Keywords:** synucleinopathy, Parkinson’s disease, histone acetyltransferase, α-synuclein

## Abstract

α-synuclein (αS) is a β-sheet intracellular protein that has been implicated as a major pathological hallmark of Parkinson’s disease (PD). Several studies have shown that overexpression of αS causes dopaminergic cell loss; however, the role of αS in apoptosis remains not fully known. Therefore, this study aims to address the mechanisms of the αS overexpression model in apoptosis and to its correlation with PD pathogenesis. Here, we used a human αS (hαS) plasmid to characterize the role of ectopic αS in neuronal apoptosis in sporadic PD *in vitro*. We found that overexpression of αS transcriptionally upregulated Bim-mediated apoptosis in neuronal SH-SY5Y cells. Interestingly, αS overexpression inhibited general control non-depressible 5 (GCN5), a histone acetyltransferase (HAT), and promoted transcriptional upregulation of Bim. Consequently, co-overexpression of GCN5 in the αS overexpressed model showed a reversal of αS toxicity in neuronal cells. These *in vitro* findings support the hypothesis of αS-mediated histone deacetylation and dopaminergic neuronal loss in PD. Moreover, our study indicates that therapeutic activation/homeostasis of GCN5 may benefit PD and other α-synucleinopathies.

## INTRODUCTION

Parkinson’s disease (PD) is the second most prevalent disease among people over 65 years of age, affecting at least 1% of the population [1, 2]. Lewy pathology, α-synuclein--rich proteinaceous cytoplasmic inclusions, is a histological hallmark of clinical PD and other synucleinopathy [3–5]. α-synuclein (αS) is a cytosolic intracellular protein [6] that is normally expressed at the presynaptic terminals. Several studies have noted nuclear localization of this protein in experimental models [7–9] and in patients with multiple system atrophy, a distinct form of synucleinopathy [10, 11]. One study has shown the physiological role of αS at the nucleus and mechanistically reported the role of the histone-αS complex in neurotoxicity [12]. However, there is a fundamental question that remains unanswered: does αS form any complex with endogenous histone acetyltransferase (HAT).

The kinetic balance between HAT and histone deacetylase (HDAC) activities regulates the steady acetylation of histone and non-histone proteins [13], resulting in cellular homeostasis. This balance also regulates cellular fate [14] by regulating different gene expressions and repression. Loss of neuronal homeostasis by losing the HAT: HDAC balance has been viewed as being related to neuronal apoptosis and neurodegeneration [15, 16]. In addition, loss of acetylation is also related to the loss of important HAT members—for example, the general control non-derepressible 5 (GCN5) [17]—resulting in apoptosis. GCN5 was the first identified enzyme with intrinsic HAT activities that is also capable of linking histone acetylation to transcriptional regulation [18, 19]. Supporting the neuroprotective role of GCN5, a study showed that GCN5-mediated acetylation of peroxisome proliferator-activated receptor γ coactivator-1α (PGC-1α), protects neuronal cells against MPP^+^-induced oxidative stress [20]. GCN5^−/−^ mice died during embryogenesis due to a combination of excessive apoptosis [21, 22] and loss of GCN5 transcriptionally upregulated BH3-only protein (Bim) and caspase-dependent neuronal apoptosis [17].

Although the histone-αS complex formation and its role in neurotoxicity are known, the interaction of αS with GCN5 has yet to be discovered. In this study, we investigated the involvement of GCN5 in synucleinopathy. To do so, we used (human) αS-transfected neuroblastoma (SH-SY5Y) cells, in which αS disrupted cell viability. Further, we found that overexpression of αS interferes with the GCN5 basal level, which we predicted could play a critical role in αS-mediated neurotoxicity. Our effort to understand αS-GCN5 interaction has found important pathogenesis of α-synucleinopathy and PD.

## RESULTS

### Overexpression of αS reduces cell viability

**Figure 1:**
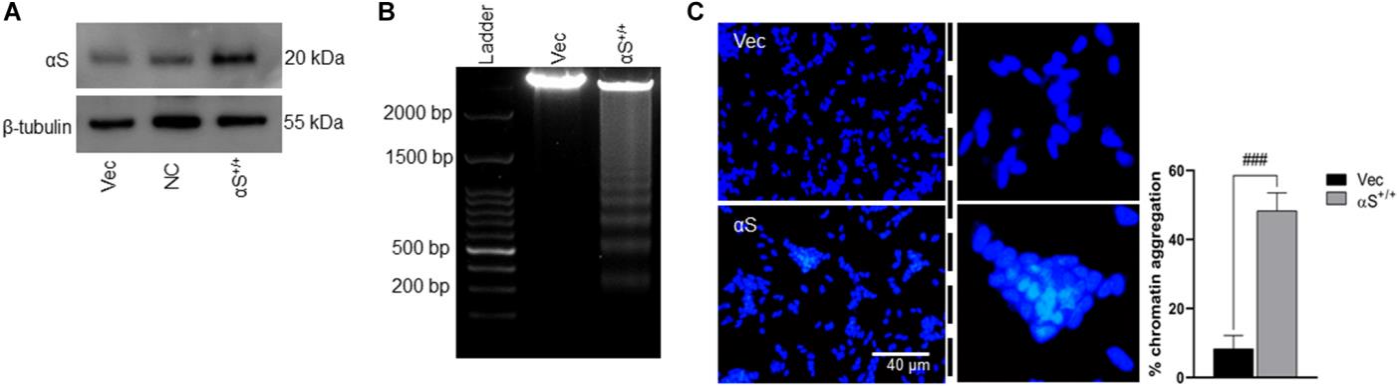
**αS overexpression and toxicity.** (**A**) SH-SY5Y neuroblastoma cells were transfected by human αS plasmid. (**B**) SH-SY5Y cells were transiently transfected by αS, and post-transfection incubated for an additional 24 h and subjected to test cell viability by DNA laddering and nucleic staining by DAPI (**C**) where the whitish inclusions (cropped) indicated apoptotic cells with nuclear pyknosis; the pyknosis rate (% chromatin aggregation) was calculated by the scoring percent of cells with nuclear pyknosis in total DAPI-stained cells (scale bar = 40 μm). ^###^*p* < 0.001 compared between vector (vec) vs. αS^+/+^.

Several studies indicated that ectopic αS is a major hallmark of neuronal cell death in PD pathology [23] and that αS overexpression promotes cell death via changing sub-cellular localization [12]. Therefore, we investigated whether our αS (human) plasmid overexpression (Figure 1A) affected the viability of SH-SY5Y neuroblastoma (Figure 1B, 1C). We used DNA fragmentation (DNA ladder) and DAPI staining assay of transiently transfected vectors or αS plasmids. After 48 hours of transfection, αS showed significant chromatin aggregation and increased DNA fragmentation. αS at the nucleus masks H3 histone and inhibits H3 activities at the chromatin level [12]. Also, overexpression of sporadic or familial (A53T and A30P) αS phosphorylates at ser129 reduces cell viability via aggregation [24]. Corroborating previous findings, our data indicate that ectopic αS regulates neuronal cell fate.

### Overexpression of αS activates bim-mediated apoptotic signaling

**Figure 2:**
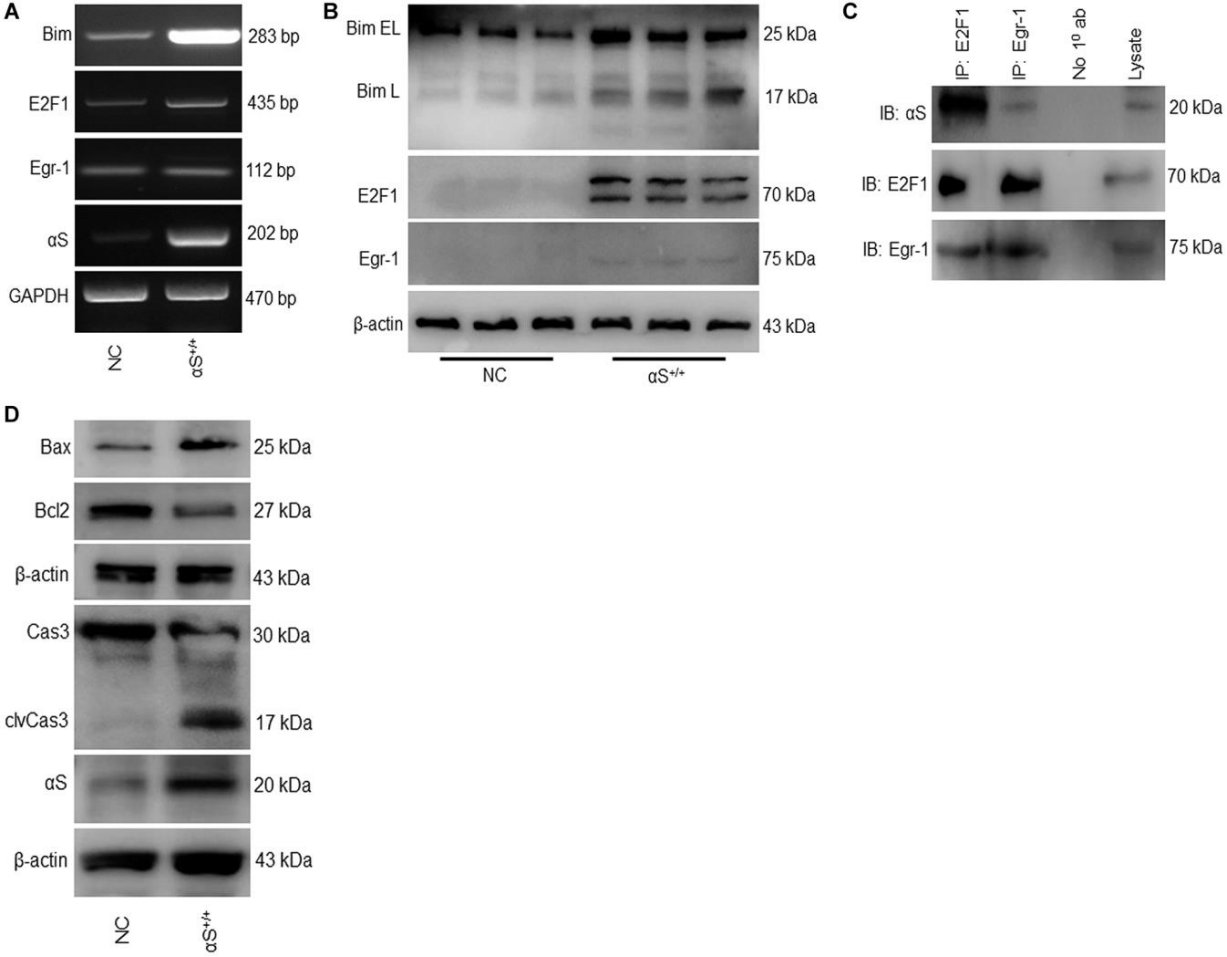
**Overexpression of αS promotes Bim-mediated apoptotic pathway.** (**A**) mRNA and (**B**) protein expression of Bim and its transcription factors in non-transfected cells (NC) and transfected (αS^+/+^) cells. (**C**) SH-SY5Y cells were co-immunoprecipitated by anti-E2F1 and anti-Egr1 or A/G beads only (No 1^0^ ab) as negative control and probed for anti-αS. (**D**) Protein expression of apoptotic markers.

The BH3-only protein Bim is a pro-apoptotic protein that has been previously shown to upregulate during apoptosis. Bim induces apoptosis by binding to Bcl-2 or Bcl-xl and antagonizes their anti-apoptotic functions. In the αS^+/+^ model, both the mRNA and protein levels of Bim increased substantially (Figure 2A, 2B). A previous study demonstrated that Bim genes are transcriptionally regulated by Egr-1 and E2F1 [17, 25]. Therefore, we tested the impact of αS overexpression on Bim transcriptional factors E2F1 and Egr-1 (Figure 2A, 2B). We found that, in both mRNA and protein levels, overexpression of αS significantly upregulated E2F1 but not Egr-1. This was further corroborated by co-immunoprecipitating both transcription factors in non-transfected SH-SY5Y cells (Figure 2C). Immunoprecipitation showed that anti-E2F1 endogenously precipitated anti-αS more intensely than anti-Egr-1. Moreover, Bim is an upstream regulator of the caspase-3-mediated apoptotic signaling pathway. αS overexpression increases the Bim level that inhibits Bcl-2, frees Bax, and activates caspase-3 (Figure 2D).

### αS Inhibits endogenous GCN5 expression

**Figure 3:**
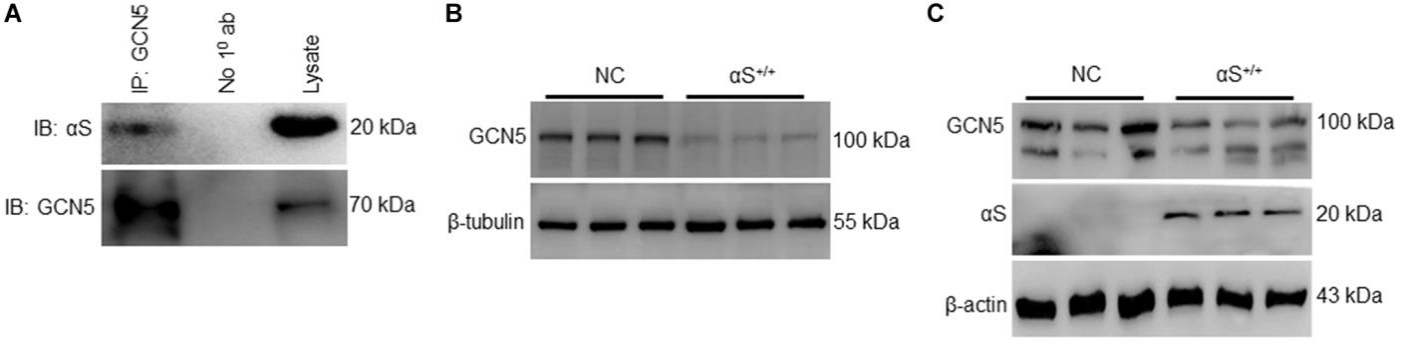
**αS^+/+^ inhibiting endogenous GCN5 and inhibiting GCN5 expression.** (**A**) co-immunoprecipitation of GCN5 in wildtype SH-SY5Y and probed for anti-αS. Endogenous protein expression of GCN5 in neuronal SH-SY5Y cells (**B**) and non-neuronal HEK293T cells (**C**) transfected (αS) or NC.

A recent study reported that GCN5 transcriptionally regulates Bim and downstream caspase-3 activation [17]. Though αS interacts and inhibits neuronally expressed acetyltransferase enzymes CBP, p300, and P/CAF [12, 26], it is generally unknown whether αS and GCN5 endogenously interact. To confirm endogenous binding, we co-immunoprecipitated GCN5 in non-transfected SH-SY5Y cells and probed anti-αS (Figure 3A). Furthermore, we evaluated the impact of αS overexpression on endogenous GCN5 expression in neuronal SH-SY5Y (Figure 3B) and non-neuronal HEK293T cells (Figure 3C). A significant decrease, compared to usual, in GCN5 expression in αS^+/+^ indicates that GCN5 may be involved in αS-mediated Bim upregulation.

### Inhibition of GCN5 triggers αS overexpression-mediated bim upregulation

**Figure 4:**
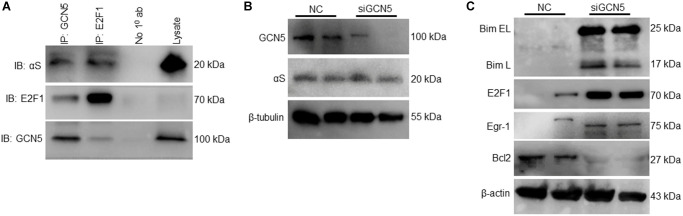
**Inhibition of GCN5 by αS triggering Bim expression.** (**A**) Co-immunoprecipitation of GCN5 and E2F1 in neuronal SH-SY5Y cells and probed for anti-αS. (**B**) GCN5 siRNA was used to knockdown GCN5 in SH-SY5Y cells; protein lysates were collected 24 h after siGCN5 induction and probed for anti-GCN5 and anti-αS. (**C**) Protein expression of apoptotic and anti-apoptotic markers.

As we found αS^+/+^ inhibits GCN5 and regulates pro-apoptotic Bim, we tested the hypothesis that GCN5 is involved in Bim’s transcriptional regulation. To test this hypothesis, we co-immunoprecipitated GCN5 and E2F1 and probed anti-αS (Figure 4A). Since the loss of GCN5 upregulates both transcription factors of Bim [17], our results indicate that ectopic αS interacts and inhibits GCN5 and that this upregulates the E2F1 level. Next, we checked whether the knockdown of GCN5 by siRNA affects endogenous αS expression (Figure 4B); however, no significant changes were observed. siGCN5 substantially upregulates Bim, Egr-1, and E2F1 levels but decreases Bcl2 expression in neuronal cells (Figure 4C). These data suggest that GCN5 could be a potential target to reduce neuronal apoptosis in PD and other synucleinopathy.

### Targeting GCN5 ameliorates αS-mediated apoptosis

**Figure 5:**
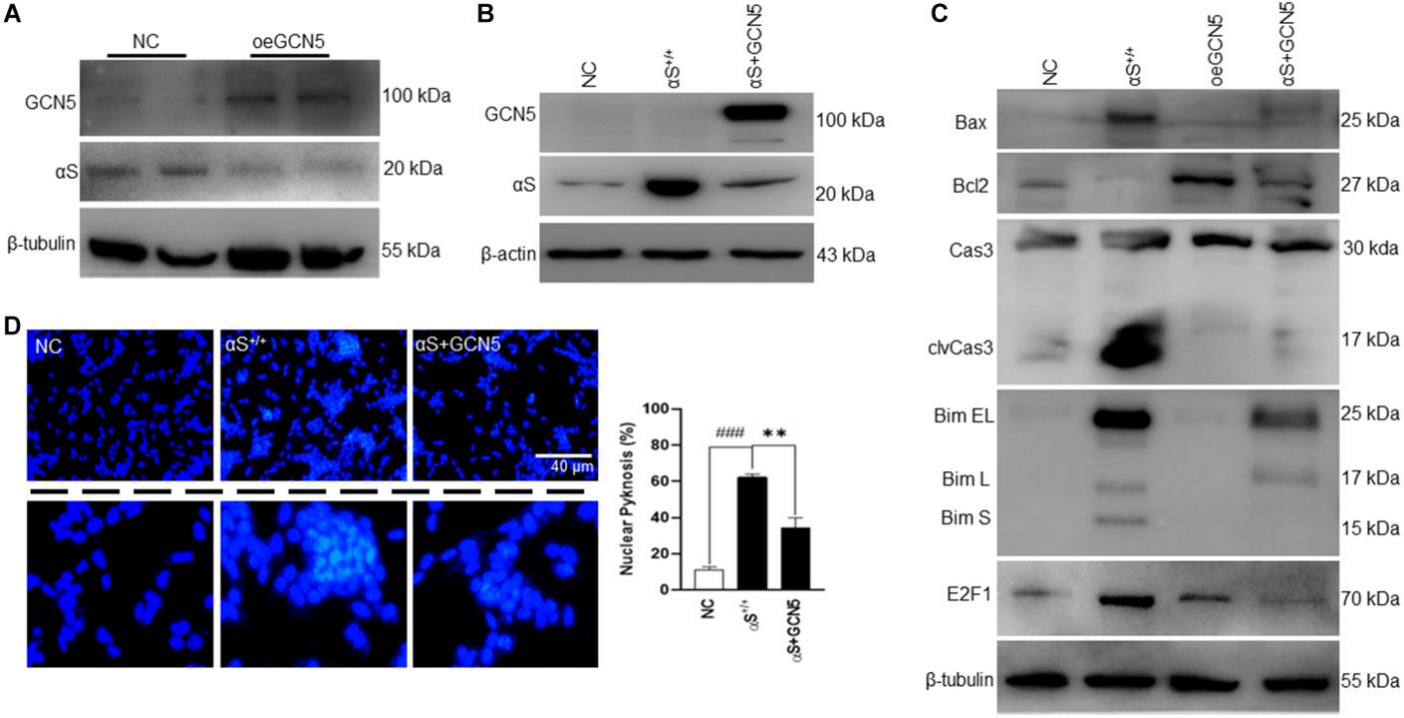
**Co-overexpression of αS-GCN5 inhibits apoptotic neuronal cell death.** (**A**) Endogenous protein expression of αS in GCN5 overexpressed (oeGCN5) or NC cells. (**B**) Protein was isolated from NC, αS^+/+^ and αS+GCN5 cells and probed for specific proteins. (**C**) Protein expression of apoptotic markers using lysates from NC, αS^+/+^, oeGCN5 and αS+GCN5 cells. (**D**) Nucleic staining by DAPI where the whitish inclusions (cropped) indicated apoptotic cells with nuclear pyknosis; the pyknosis rate (% chromatin aggregation) was calculated by the scoring percent of cells with nuclear pyknosis in total DAPI-stained cells (scale bar = 40 μm). ^###^*p* < 0.001 compared between vector (vec) vs. αS^+/+^ and ^**^p < 0.01 compared between αS^+/+^ vs. αS+GCN5.

Until now, this study has indicated that αS overexpression dismantles GCN5 homeostasis, which triggers apoptosis in neuronal cells. Therefore, we hypothesized that GCN5 homeostasis by co-activation could benefit PD patients by ameliorating apoptosis. First, we tested whether GCN5 overexpression (oeGCN5) modulates αS endogenous levels (Figure 5A) and found that oeGCN5 downregulated endogenous αS expression. Later, we co-overexpressed αS and GCN5 (αS+GCN5) in SH-SY5Y cells (Figure 5B**)** and tested for an apoptotic marker expression (Figure 5C). The co-overexpression of αS and GCN5 were found to substantially reduce transcriptional upregulation of Bim and improve Bcl2 levels, which reduced caspase-3 activation. Subsequently, co-overexpression downregulated neuronal cell death (Figure 5D).

## DISCUSSION

Multiplications of the *SNCA* gene locus showed autosomal dominant PD, where gene dose was the determinant of severity and onset. This indicates that αS mutations, which alter protein output, are not the determinant of αS pathology—wild-type αS overexpression could also cause the disease. Moreover, overexpression of αS, due to either increased expression or reduced clearance, is a common feature of a sporadic disease condition. Since the first identification of αS, most related studies have focused on αS roles in nerve terminals [27, 28]; however, a definitive role is yet to be found. Nevertheless, some studies have shown that the nuclear localization of αS [7, 8, 12, 29] contributes to its cytotoxic role [12]. In this study, we observed αS immunoreactivity with the important HAT GCN5. We also found that αS suppressed GCN5 endogenous levels in dopaminergic neuronal cells and loss of GCN5 promoted Bim-mediated apoptotic signaling.

Bim is a major regulator of the intrinsic apoptosis pathway under both physiological and pathological conditions. Pro-activities and expression of Bim are tightly regulated by several transcription factors, including Fox3a, E2F1, c-Myc, and Egr-1 [30]. Ectopic E2F1 has shown upregulation of Bim expression and neuronal apoptosis [31]. Also, histone deacetylases (HDAC) have been observed targeting the transcription factor E2F1 in the hyperactivation of Bim and apoptosis [32]. αS plays as critical a role as HDAC, which we also demonstrated in the present work. Overexpression of αS activated E2F1 and significantly upregulated Bim expression. In addition, Bim could induce apoptosis directly or indirectly by neutralizing anti-apoptotic Bcl2 and promoting Bax [30]. This indicates that αS overexpression upregulates Bim levels, thus binding Bcl2 and masking Bcl2 anti-apoptotic activities.

In the nucleus, αS inhibits H3 histone acetylation [12], promoting neurotoxicity. HAT members—for example, GCN5—are important for maintaining the precious HAT: HDAC balance. Loss of the basal level of HAT might promote histone deacetylation, thus impairing acetylation homeostasis. Moreover, a recent study characterized the anti-apoptotic role of GCN5 [17, 20], which also indicates that inhibition of GCN5 activities could trigger apoptosis. However, until this study, there have been no reports of the direct inhibition of GCN5 by αS. In this study, we found that GCN5 and αS have endogenous protein-protein interaction and that αS^+/+^ downregulates the basal level of GCN5. Several studies have reported that HAT members modulate neuronal fate: for example, enhanced p3000 HAT activity upregulated PKCδ-mediated neuronal apoptosis [26], and increased Tip60 HAT activities induced APP-mediated apoptosis in Alzheimer’s disease [33]. While the loss of HAT activities promotes apoptosis, loss of the CREB-binding protein (CBP) HAT activity promotes caspase-6-mediated apoptosis [34], and inhibition by MB-3 or knockdown of GCN5 induces Bim-transcription and apoptosis [17]. Our findings indicate that, among the other neurotoxic pathways of αS, GCN5 activity inhibition is involved. We also correlated that loss of GCN5 via αS overexpression enhanced transcription factor E2F1 and activated Bim. Furthermore, αS downregulates anti-apoptotic Bcl-2, frees Bim protein that activates Bax, and permeabilizes the mitochondrial membrane, resulting in caspase-dependent apoptosis [35].

αS has been reported to inhibit multiple HAT activities, but there has been no previous research on GCN5 activities in this context. However, loss of GCN5, but not p300 and Tip60, was found to upregulate apoptosis [17]. Here, we report a protective role of GCN5 in α-synucleinopathy. Co-overexpression of GCN5 rescues neuronal cells via downregulating Bim transcription and reversing caspase-3-mediated apoptosis.

**Figure 6:**
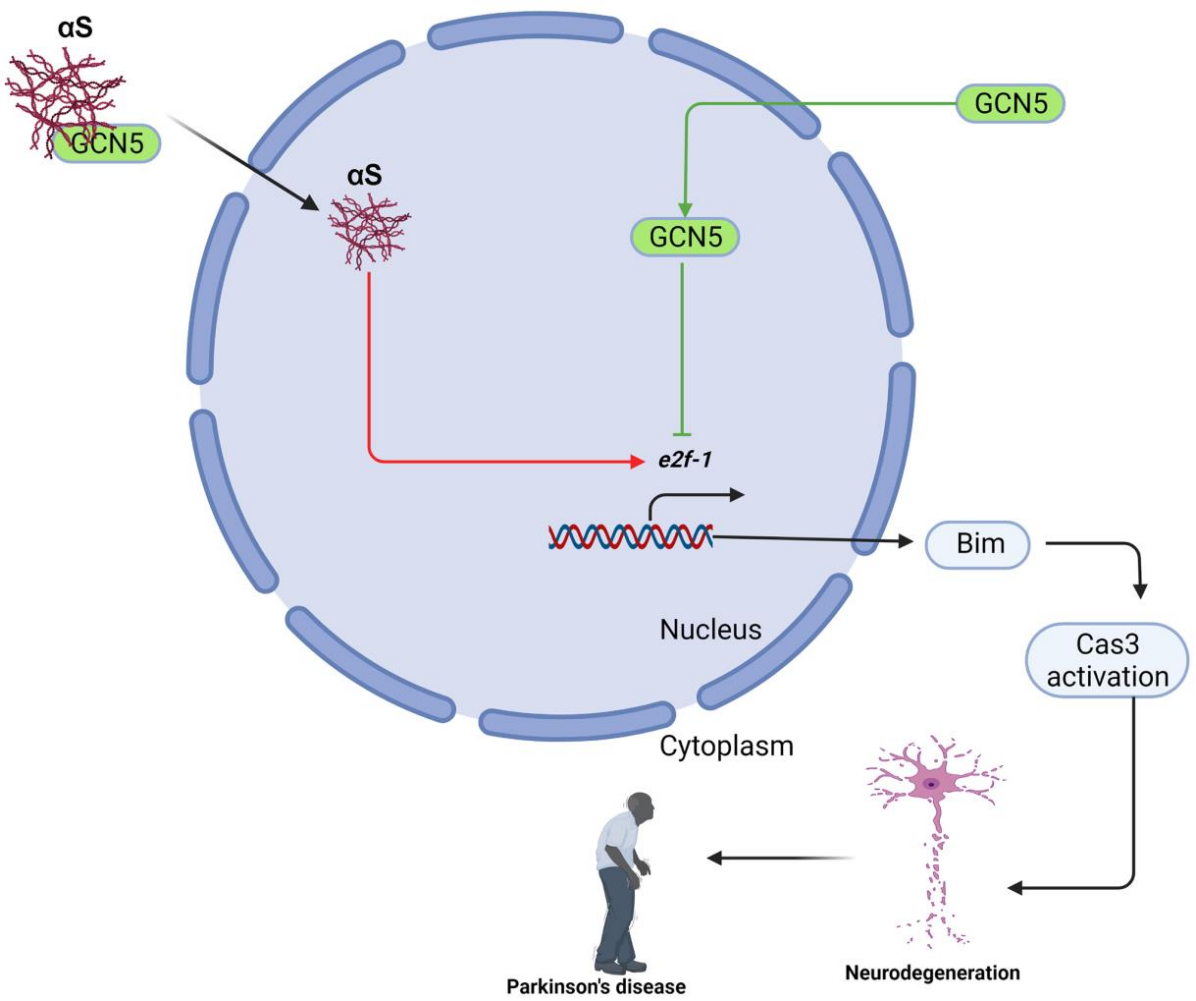
Schematic presentation of αS-GCN5 role in neuronal cell apoptosis.

In conclusion, we showed a new role of αS overexpression in apoptosis. We also found that αS interacts with and inhibits GCN5 and loss of GCN5 promotes Bim-mediated apoptosis in neuronal cells (Figure 6). Promisingly, HDAC inhibitors have shown anti-apoptotic activities in many neurodegenerative diseases by preventing histone deacetylation. Yet, we found a new pathway in which the development of the GCN5 activator would build HAT: HDAC homeostasis and could reverse neuronal apoptosis in Parkinson’s disease pathogenesis. Since αS acts in the nucleus to promote neurotoxicity, mostly by interfering with histone acetylation and HAT member activities, our findings suggest that the development of GCN5-dependent therapeutics might benefit PD and other synucleinopathy.

## METHODS

### Cell culture and treatment

Human neuroblastoma SH-SY5Y cells were obtained from the American Type Culture Collection (ATCC; Manassas, VA, USA). The neuroblastoma cells were cultured in DMEM/F12 (1:1) supplemented with 100 U/mL penicillin/streptomycin and 10% (v/v) inactivated FBS in an incubator with the conditions maintained at 37°C and 5% CO_2_. The cells were trypsinized (0.05% trypsin-EDTA) after reaching 80–90% confluence for sub-culture, and the media was replaced every 2 days. Each experiment was conducted at least three times from three consecutive passages for statistical analysis.

### Lentiviral transfection

The lentiviral plasmid *SNCA* (pLenti-Glll-CMV-C-term-HA; cat# LV315044), KAT2A (pLenti-Glll-CMV-C-term-HA; cat# LV194350) and empty vector (pLenti-lll-Blank; cat# LV587) were obtained from Applied Biological Materials Inc. (Crestwood Place, BC, Canada). After transforming into *E. coli* (DH5α), the large colonies were picked for further amplification and purification using the PureLink plasmid filter kit (Thermo Fisher Scientific, V.A., Lithuania), as per the manufacturer’s instructions. The empty vector served as a control. Amplified and purified plasmids were infected into 70–80% confluent SH-SY5Y cells using a Lipofectamine 3000 (cat# L3000015; Thermo Fisher Scientific) reagent. Post-transfection, cells were kept for 48 hours and then treated with 0.75–1 μg/ml puromycin to select stably transfected cells. The stably transfected αS, GCN5, and Vec were subjected to WB and reverse transcription-PCR (RT-PCR) for further analysis.

### Reverse transcription-PCR (RT-PCR)

The total RNA was extracted and isolated using the TRIzol reagent (Invitrogen, Carlsbad, CA, USA) as described by the supplier. We used the following primers specific for:

- Bim (forward 5′-CTACCAGATCCCCACTTTTC-3′, reverse 5′-GCCCTCCTCGTGTAAGTCTC-3′);
- E2F1 (forward 5′-GACTGTGACTTTGGGGACT-3′, reverse 5′-TGTTCACCTTCATTCCC-3′);
- Egr-1 (forward 5′-CGAGCGAACAACCCTACGAGC-3′, reverse 5′-GAGGCAGAGGAAGACGATGAAGC-3′);
- αS (forward 5′-AGTGGCTGAGAAGACCAAAG-3′, reverse 5′-GTCAGGATCCACAGGCATATC-3′);
- GCN5 (forward 5′-AGCGGTTGTTCCCAGCACCC-3′, reverse 5′-GACAGCACAGAAGACAATCT-3′);
- GAPDH (forward 5′-GCAAAGTGGAGATTGTTGCCATC-3′ and reverse 5′-CATATTTCTCGTGGTTCACACCC-3′).

Photos were taken in a Davinch-K Gel Imaging System (MC2000/CG550, Youngin Lab Plus Co., the Republic of Korea).

### Western blot analysis

The cells were washed twice with cold PBS and subjected to lyse using a lysis buffer (1× RIPA lysis buffer containing a protease and phosphatase inhibitor (1:1) cocktail). Whole mixtures were centrifuged at 14,000 rpm at 4°C for 15 minutes, and the supernatants were collected carefully without disturbing the pellets. The total protein obtained from cell lysates was quantified using a DC™ protein assay kit (Bio-Rad, USA) according to manufacturer instructions. Equal amounts of protein (20 μg) were separated electrophoretically using 8–15% sodium dodecyl sulfate-polyacrylamide electrophoresis (SDS-PAGE) gel and then transferred onto polyvinylidene-difluoride (PVDF) membranes (Millipore, Bedford, MA, USA). The membranes were incubated at room temperature for 1 hour with 3% bovine serum albumin in Tris-buffered saline (containing 0.1% Tween 20 buffer) to prevent nonspecific binding. The blots were then incubated overnight at 4°C with specific primary antibodies, including anti-Caspase3 (#H-277; Santa Cruz Biotechnology), anti-clvCaspase3 (#9661; cell signaling technology), anti-alpha-synuclein (#ab212184; Abcam), anti-alpha-synuclein LB509 (#ab27766; Abcam), anti-alpha-synuclein (211) (#sc-12767; Santa Cruz Biotechnology), anti-EGR-1 (#4154; cell signaling technology), anti-E2F1 (#3742; cell signaling technology), anti-Bim (#2819; cell signaling technology), anti-GCN5 (#sc-365321; Santa Cruz Biotechnology), anti-GCN5 (#MA45-14886; Invitrogen) anti-Bcl-2 (#N-19; Santa Cruz Biotechnology), anti-Lamin B1 (#12586; cell signaling technology), anti-Ac Histone H3 (#sc-56616; Santa Cruz Biotechnology), anti-Histone H3 (#sc-517576; Santa Cruz Biotechnology), anti-Ac Lys (#AB3879; Sigma-Aldrich) at 1:1000, anti-β-actin (#C4; Santa Cruz Biotechnology), and anti-β-tubulin (#2146; cell signaling technology) at 1:5000 concentration. Next, each blot was incubated at room temperature with either an anti-mouse or anti-rabbit (1:10,000) secondary antibody. The blots were visualized with an enhanced chemiluminescence detection system (LAS 500; GE Healthcare Bio-Sciences AB, Sweden) per the recommended protocol.

### DAPI staining

SH-SY5Y or transfected cells were grown to a confluency of 70–80% then washed with cold PBS once, followed by fixation with 4% cold PFA for 15 minutes at room temperature. After discarding PFA, cells were washed twice and permeabilized with 0.1% Triton X-100 for 10 minutes at room temperature. Cells were stained with DAPI (2 μg/ml) for 10 minutes at room temperature and images were immediately captured using a Nikon Eclipse Ts2R fluorescence microscopy system and were processed by NIS-Elements BR-2.01.00 software (which came with the instrument).

### RNA interference

siRNA of GCN5 (#sc-37946; Santa Cruz Biotechnology) was used. The interference efficiency was detected by comparing siRNAs with the normal control (NC). Each siRNA was transfected into SH-SY5Y cells by RNAiMax (Invitrogen) according to the manufacturer’s protocol. Forty-eight hours after transfection, cell lysates were harvested and processed for WB to detect the expression of these proteins.

### Co-immunoprecipitation

The SH-SY5Y cells were seeded at 5 × 10^5^ density for 48 hours in a P100 plate and lysed in a non-denaturing RIPA buffer for 1 hour of incubation at 4°C on a wheel. After centrifugation, the supernatant was quantified with a DC™ protein assay kit (Bio-Rad, USA), and 0.6 mg of total protein extract was added to 50 μL of A/G agarose containing anti-GCN5, anti-E2F1, and anti-Egr-1 O/N at 4°C (rotation). The next day, the same binding buffer was used to perform four washes by centrifugation at 11,000 × g for 1 minute. The precipitated agarose was finally boiled and used for a western blot with the respective antibodies.

### Statistical analysis

Statistical analyses were performed using GraphPad Prism (version 8.0.1; La Jolla, CA, USA) software. Data are represented as means ± SEM (standard error mean) of the three independent experiments. Student’s two-tailed *t*-test was used for comparison of the two different groups, and a one-way ANOVA, followed by Sidak’s multiple comparisons, was used for analysis more than two groups. A *p*-value of <0.05 was considered statistically significant.

### Data availability

The datasets generated and/or analyzed during the current study are publicly available upon acceptance of this manuscript.
